# Supplementary material for: Integrating Transcriptomics, Proteomics, and Metabolomics to Investigate the Mechanism of Fetal Placental Overgrowth in Somatic Cell Nuclear Transfer Cattle
Source: Int J Mol Sci. 2024 Aug 29;25(17):9388. doi: 10.3390/ijms25179388 (PMC11395630; doi:10.3390/ijms25179388)
Supplement: Supplementary file 1 [file ijms-25-09388-s001.zip › ijms-3157191-supplementary.pdf]

Table S1. Top 10 GO terms up genes.

GO ID	GO Term	GO Category	Q-Value
GO:0032964	Biological Process	collagen biosynthetic process	$2.29x10^{-7}$
GO:0032963	Biological Process	collagen metabolic process	$4.28x10^{-7}$
GO:0030199	Biological Process	collagen fibril organization	$4.79x10^{-7}$
GO:0060070	Biological Process	canonical Wnt signaling pathway	$4.79x10^{-7}$
GO:0007229	Biological Process	integrin-mediated signaling pathway	$4.90x10^{-7}$
GO:0031589	Biological Process	cell-substrate adhesion	$6.93x10^{-7}$
GO:0030198	Biological Process	extracellular matrix organization	$7.18x10^{-7}$
GO:0043062	Biological Process	extracellular structure organization	$1.10x10^{-6}$
GO:0097435	Biological Process	supramolecular fiber organization	$1.26x10^{-6}$
GO:0043087	Biological Process	regulation of GTPase activity	$1.41x10^{-6}$

Table S2. Top 10 GO terms down genes.

GO ID	GO Term	GO Category	Q-Value
GO:0070646	Biological Process	protein modification by small protein removal	$1.81x10^{-6}$
GO:0016070	Biological Process	RNA metabolic process	$4.08x10^{-6}$
GO:0016192	Biological Process	vesicle-mediated transport	$4.19x10^{-6}$
GO:0046907	Biological Process	intracellular transport	$4.95x10^{-6}$
GO:0051716	Biological Process	cellular response to stimulus	$5.02x10^{-6}$
GO:0033554	Biological Process	cellular response to stress	$5.15x10^{-6}$
GO:0090304	Biological Process	nucleic acid metabolic process	$5.40x10^{-6}$
GO:0045935	Biological Process	positive regulation of nucleobase-containing compound metabolic process	$5.50x10^{-6}$
GO:0046483	Biological Process	heterocycle metabolic process	$5.86x10^{-6}$
GO:0051173	Biological Process	positive regulation of nitrogen compound metabolic process	$5.87x10^{-6}$

Table S3. Top 10 KEGG up genes.

Pathway ID	Pathway Name	Q-Value
map04974	Protein digestion and absorption	$3.18x10^{-8}$

map04512	ECM-receptor interaction	$3.34x10^{-7}$
map04510	Focal adhesion	$3.06x10^{-6}$
map04810	Regulation of actin cytoskeleton	$1.95x10^{-5}$
map05225	Hepatocellular carcinoma	$3.36x10^{-5}$
map00010	Glycolysis Gluconeogenesis	$7.26x10^{-5}$
map04380	Osteoclast differentiation	$6.97x10^{-5}$
map05217	Basal cell carcinoma	$1.08x10^{-4}$
map04662	B cell receptor signaling pathway	$1.60x10^{-4}$
map04145	Phagosome	$1.51x10^{-4}$

Table S4. Top 10 KEGG down genes.

Pathway ID	Pathway Name	Q-Value
map05168	Herpes simplex virus 1 infection	$9.24x10^{-9}$
map04140	Autophagy - animal	$8.78x10^{-5}$
map04214	Apoptosis - fly	$1.49x10^{-4}$
map04010	MAPK signaling pathway	$2.11x10^{-4}$
map04013	MAPK signaling pathway - fly	$5.25x10^{-4}$
map04923	Regulation of lipolysis in adipocytes	$5.10x10^{-4}$
map05418	Fluid shear stress and atherosclerosis	$8.15x10^{-4}$
map04668	TNF signaling pathway	$9.34x10^{-4}$
map04935	Growth hormone synthesis, secretion and action	$1.09x10^{-3}$
map04611	Platelet activation	$1.31x10^{-3}$

Table S5. Top 10 GO up proteins.

GO ID	GO Term	GO Category	Q-Value
GO:0030018	Cellular Component	Z disc	$1.32x10^{-4}$
GO:0061061	Biological Component	muscle structure development	$1.49x10^{-4}$
GO:0004888	Molecular Function	transmembrane signaling receptor activity	$5.98x10^{-4}$
GO:0031226	Cellular Component	intrinsic component of plasma membrane	$7.56x10^{-4}$

GO:0005886	Cellular Component	plasma membrane	$1.27x10^{-3}$
GO:0005887	Cellular Component	integral component of plasma membrane	$1.54x10^{-3}$
GO:0016021	Cellular Component	integral component of membrane	$1.80x10^{-3}$
GO:0060089	Molecular Function	molecular transducer activity	$1.94x10^{-3}$
GO:0038023	Molecular Function	signaling receptor activity	$1.94x10^{-3}$
GO:0031224	Cellular Component	intrinsic component of membrane	$2.12x10^{-3}$

Table S6. Top 10 GO down proteins.

GO ID	GO Term	GO Category	Q-Value
GO:0005777	Cellular Component	peroxisome	$4.45x10^{-4}$
GO:0004866	Molecular Function	endopeptidase inhibitor activity	$4.62x10^{-4}$
GO:0030414	Molecular Function	peptidase inhibitor activity	$9.01x10^{-4}$
GO:0042579	Cellular Component	microbody	$9.49x10^{-4}$
GO:0061135	Molecular Function	endopeptidase regulator activity	$1.24x10^{-3}$
GO:0044281	Biological Component	small molecule metabolic process	$1.38x10^{-3}$
GO:0005615	Cellular Component	extracellular space	$1.55x10^{-3}$
GO:0006629	Biological Component	lipid metabolic process	$6.49x10^{-3}$
GO:0098590	Cellular Component	plasma membrane region	$7.73x10^{-3}$
GO:0061134	Molecular Function	peptidase regulator activity	$1.34x10^{-2}$

Table S7. Top 10 KEGG up proteins.

Pathway ID	Pathway Name	Q-Value
bta04510	Focal adhesion	$8.57x10^{-5}$
bta04810	Regulation of actin cytoskeleton	$4.04x10^{-4}$
bta04060	Cytokine-cytokine receptor interaction	$5.49x10^{-4}$
bta04514	Cell adhesion molecules	$6.06x10^{-4}$
bta04640	Hematopoietic cell lineage	$3.79x10^{-3}$
bta04380	Osteoclast differentiation	$1.36x10^{-2}$
bta04670	Leukocyte transendothelial migration	$1.63x10^{-2}$
bta04145	Phagosome	$4.69x10^{-2}$

bta05205	Proteoglycans in cancer	$6.96x10^{-2}$
bta04512	ECM-receptor interaction	$7.96x10^{-2}$

Table S8. Top 10 KEGG down proteins.

Pathway ID	Pathway Name	Q-Value
bta04146	Peroxisome	$5.70x10^{-3}$
bta04142	Lysosome	$1.12x10^{-2}$
bta00120	Primary bile acid biosynthesis	$4.48x10^{-2}$
bta01100	Metabolic pathways	$4.83x10^{-2}$
bta00561	Glycerolipid metabolism	$1.04x10^{-1}$
bta04913	Ovarian steroidogenesis	$1.66x10^{-1}$
bta04927	Cortisol synthesis and secretion	$1.85x10^{-1}$
bta00330	Arginine and proline metabolism	$1.96x10^{-1}$
bta00071	Fatty acid degradation	$2.59x10^{-1}$
bta00380	Tryptophan metabolism	$2.59x10^{-1}$
